# Ileosigmoidal knotting: a case report and literature review of a rare cause of acute abdomen in children

**DOI:** 10.1093/jscr/rjae142

**Published:** 2024-03-12

**Authors:** Dipak K Yadav, Sanjay K Shah, Saurav Poudel, Bivusha Parajuli, Amit Bhattarai, Dinesh Adhikari

**Affiliations:** Department of General Surgery, Nobel Medical College Teaching Hospital, Kanchanbari, Biratnagar-4, Morang 56613, Nepal; Department of General Surgery, Nobel Medical College Teaching Hospital, Kanchanbari, Biratnagar-4, Morang 56613, Nepal; Department of General Surgery, Nobel Medical College Teaching Hospital, Kanchanbari, Biratnagar-4, Morang 56613, Nepal; Department of General Surgery, Nobel Medical College Teaching Hospital, Kanchanbari, Biratnagar-4, Morang 56613, Nepal; Department of General Surgery, Nobel Medical College Teaching Hospital, Kanchanbari, Biratnagar-4, Morang 56613, Nepal; Department of General Surgery, Nobel Medical College Teaching Hospital, Kanchanbari, Biratnagar-4, Morang 56613, Nepal

**Keywords:** intestinal volvulus, intestinal obstruction, acute abdomen, sigmoid

## Abstract

Ileosigmoidal knotting (ISK) is a rare, possibly fatal cause of intestinal obstruction. ISK is a compound volvulus that is more common in Africa and Asia. ISK is mostly seen in adults, pediatric cases reported in the literature are much rarer. In this report, we present the first reported case of ISK in a pediatric patient from Nepal. An 8-year-old male child presented with symptoms of abdominal pain, vomiting, and obstipation. The abdomen was distended with generalized tenderness. Erect abdominal X-ray showed multiple air-fluid levels. Intraoperatively, gangrenous ileum loops were entangled around the sigmoid, and resection of the gangrenous ileum and sigmoid was performed. An end-to-end colo-colic anastomosis from the descending colon to the remaining sigmoid with a double-loop ileostomy was performed. Pediatric ISK is a rare fatal form of intestinal obstruction that progresses quickly to gangrene. Clinical signs and symptoms are nonspecific, making preoperative diagnosis challenging.

## Introduction

Ileosigmoidal knotting (ISK) is a rare closed-loop intestinal obstruction and a compound volvulus with a fatal outcome [1]. ISK presents as acute intestinal obstruction and is diagnosed intraoperatively [1]. Endoscopic reduction is contraindicated, and therefore ISK should be differentiated from sigmoidal volvulus [2, 3]. Parker first reported pediatric ISK in 1845 [4]. Subedi *et al*. [3] reported one adult case from Nepal in 2021.

We are presenting the first pediatric case of ISK to be reported from Nepal.

## Case report

An 8-year-old boy presented to the emergency department with 4 days of abdominal pain, initially around the paraumbilical region and later becoming generalized. He also experienced abdominal distension, obstipation, and vomiting. The physical exam revealed dehydration, tachycardia, and a distended and tender abdomen with absent bowel sounds. A nasogastric tube was inserted 300 ml of bilious content was drained. His vitals were stable.

**Figure 1:**
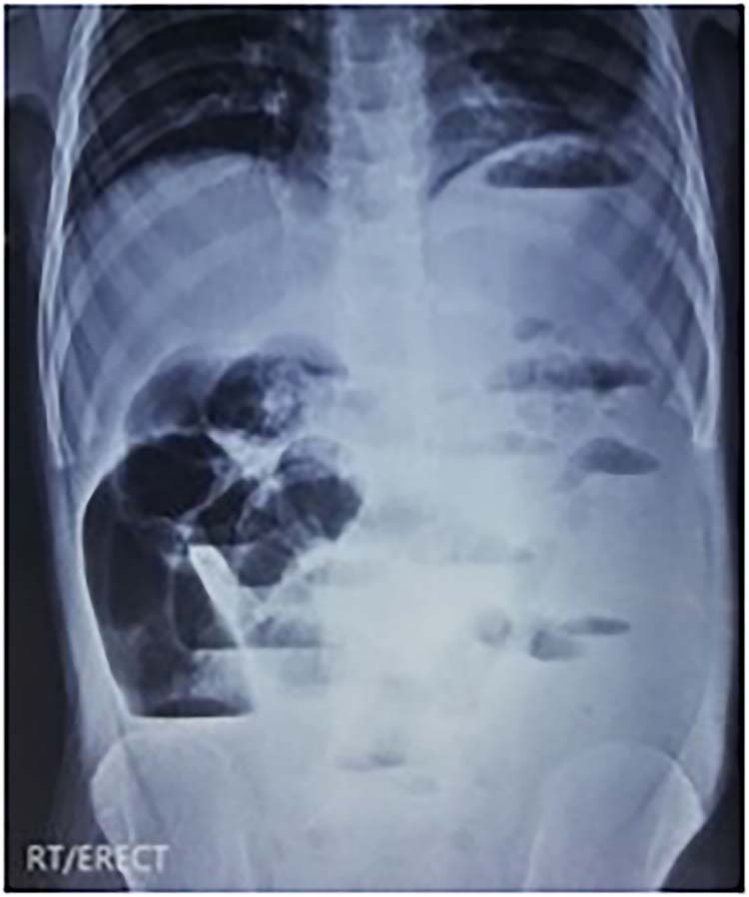
X-ray of abdomen erect view showing multiple air-fluid levels.

An erect X-ray abdomen (Fig. 1) showed multiple air-fluid levels and a distended bowel.

Lab results indicated leukocyte count of 10 200 with neutrophil 80%, hemoglobin 8gm/dL, serum sodium 121 mmol/L, and C-reactive protein 20.9 mg/L. Acute small bowel obstruction was suspected, leading to an emergency exploratory laparotomy.

**Figure 2:**
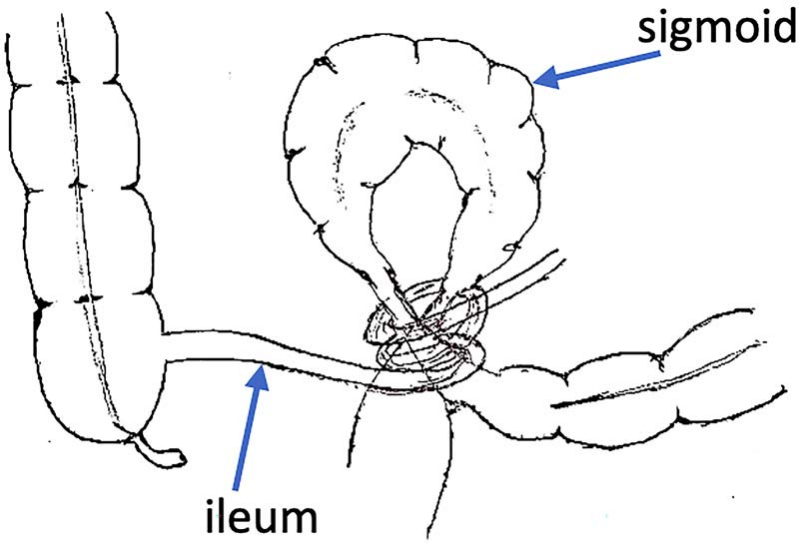
ISK.

Intraoperatively, hemorrhagic peritoneal fluid was found, along with distended and gangrenous bowel loops that were entangled together (Fig. 2).

**Figure 3:**
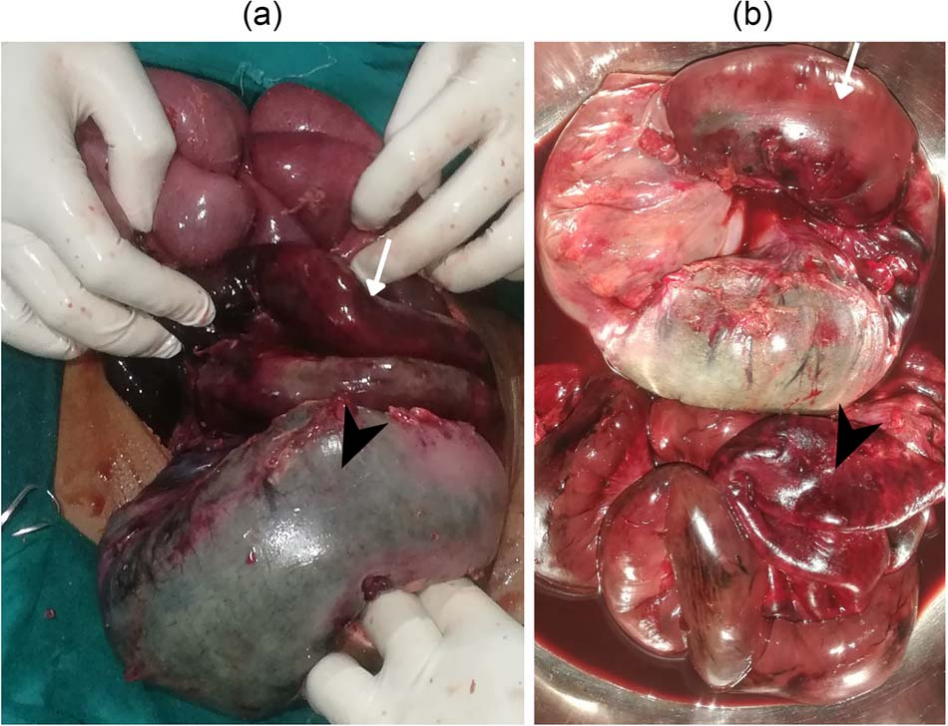
(a) Gangrenous ileum (arrow) and sigmoid (arrow head) (b) resected gangrenous ileum (arrow head) and sigmoid (arrow).

Exploration revealed a twisted knot between gangrenous sections of the ileum and sigmoid colon (Fig. 3a). After untwisting the knot, about 100 cm of ileum, around 10 cm from the ileocecal junction, and 20 cm of sigmoid were gangrenous, necessitating their resection. A double barrel ileostomy was performed, followed by an end-to-end colo-colic anastomosis between descending colon and distal sigmoid (Fig. 3b). The patient recovered well and was discharged on the 5th postoperative day.

### Follow-up event

The patient’s ileostomy functioned well during the 1-month follow-up. In the 6th postoperative week, an uneventful ileostomy reversal took place, leading to the patient’s complication-free discharge from the hospital.

## Discussion

Shepherd [5] provided a seminal description of the pathogenesis and clinical features of ISK. ISK is thought to result from a combination of factors, including a long mesentery of the small bowel, a freely mobile small bowel, a long sigmoid colon with a narrow pedicle, and a bulky diet in the presence of an empty small bowel [5–7]. Shepherd [5] in Uganda reported the occurrence of ISK in Muslims accustomed to a single large meal during Ramzan. Other causes of ISK include the ingestion of 5-hydroxy-tryptamine, Meckel’s diverticulitis, ileocecal intussusception, and floating cecum [5, 6].

ISK is mainly reported in African and some Asian countries, as well as Middle Eastern countries. The region of Eastern Anatolia in Turkey is considered an endemic area for ISK [1, 8]. However, the condition is rare in the West and other developed nations [9]. Atamanalp *et al*. [9] found that the mean age of patients was 43.9 years, with male predominance. Atamanalp [2] reported an incidence of ISK at 1.6 cases per year and 0.4 cases per 100 000 persons per year. ISK is uncommon in children, with very few cases reported [1, 10]. The youngest documented case is of a 2-week-old patient [11].

The symptoms of ISK are nonspecific and can easily be mistaken for small bowel strangulation or sigmoid volvulus [7, 10]. Since ISK is a rare condition, particularly in children, it is usually not diagnosed until laparotomy [1, 10]. However, it is crucial to distinguish it from sigmoid volvulus because endoscopic decompression can be fatal in ISK, and the condition can rapidly progress to gangrene [2, 3, 7]. ISK is characterized by a combination of symptoms associated with both small and large bowel obstruction, including pain, obstipation, abdominal distension, nausea, vomiting, tenderness, changes in bowel sounds, either hypo or hyperactive, muscular guarding, and the presence of gangrenous material in the rectum [1, 3, 5].

Radiological findings of ISK include a double closed-loop type obstruction, with multiple air-fluid levels in X-ray scans of the abdomen [3, 10]. These findings are often missed because ISK is a rare diagnosis, particularly in children [6]. Lee *et al*. [12] described two prominent and characteristic CT findings of ISK: (i) medial deviation of the distal descending colon and cecum with a beak appearance of the afferent limb of volvulus on its medial side and (ii) a “Whirl sign” formed because of the twisting of the intestine and mesentery.

ISK causes significant volume loss and absorption of toxic material from the intestinal obstruction, as well as ischemia of the bowel and gangrene, which can lead to hypovolemic or toxic shock [3, 9]. Therefore, managing a case of ISK involves first correcting fluid, electrolyte, and acid–base imbalances [9]. The use of central venous pressure monitoring is recommended, along with nasogastric decompression, parenteral nutrition, and the use of antibiotics such as cephalosporins, aminoglycosides, and metronidazole [3, 7, 8]. This should be followed by emergency laparotomy [5, 7, 8].

The definitive management of ISK in children is based on individual risk and operative findings, similar to the approach in adults [7, 8, 10]. If the bowel is gangrenous, the knot should not be untied to prevent toxin release, absorption, and perforation [5, 6, 10]. Resection anastomosis in a single or two-stage procedure with stoma placement and reversal has been recommended by several investigators [7, 9]. The most commonly performed procedures involve ileal resection, primary anastomosis, sigmoid resection, and Hartmann’s colostomy [6, 7]. For non-gangrenous bowel, detorsion or sigmoidopexy may be considered [6, 9].

The mortality risk associated with ISK ranges from 0% to 48%, whereas gangrenous cases can result in mortality rates of up to 100%, and morbidity rates between 30% and 80% [7, 9]. The toxic shock caused by the gangrenous bowel is the most common cause of death, whereas wound infection, dehiscence, anastomosis complications, stoma complications, adhesive ileus, and systemic complications are commonly observed [6, 9, 10]. Recurrence of ISK is uncommon and is most likely to occur in patients who underwent decompression [5, 8, 9]. Despite advances in surgical care, ISK still carries a poor prognosis, which can be attributed to the rarity of the condition and the lack of knowledge among surgeons about the condition [9].

## Conflict of interest statement

None declared.

## Funding

None declared.
